# Understanding the Microstructure Evolution of 8Cr4Mo4V Steel under High-Dose-Rate Ion Implantation

**DOI:** 10.3390/ma16175876

**Published:** 2023-08-28

**Authors:** Bin Miao, Jinming Zhang, Jiaxu Guo, Xinxin Ma, Liqin Wang, Xinghong Zhang

**Affiliations:** 1 State Key Laboratory of Advanced Welding and Joining, Harbin Institute of Technology, Harbin 150001, China; 2 School of Materials Science and Engineering, Harbin Institute of Technology, Harbin 150001, China; zjm980529@163.com (J.Z.); jxguo_hit@163.com (J.G.); 3 CETC Academy of Chips Technology, Chongqing 401332, China; 4 MIIT Key Laboratory of Aerospace Bearing Technology and Equipment, Harbin Institute of Technology, Harbin 150001, China; lqwang@hit.edu.cn; 5 AECC Harbin Bearing Co., Ltd., Harbin 150001, China; zhxh710@126.com

**Keywords:** 8Cr4Mo4V steel, ion implantation, high dose rate, crystallite size, Williamson–Hall method

## Abstract

In this study, the effect of microstructure under various dose rates of plasma immersion ion implantation on 8Cr4Mo4V steel has been investigated for crystallite size, lattice strain and dislocation density. The phase composition and structure parameters including crystallite size, dislocation density and lattice strain have been investigated by X-ray diffraction (XRD) measurements and determined from Scherrer’s equation and three different Williamson–Hall (W-H) methods. The obtained results reveal that a refined crystallite size, enlarged microstrain and increased dislocation density can be obtained for the 8Cr4Mo4V steel treated by different dose rates of ion implantation. Compared to the crystallite size (15.95 nm), microstrain (5.69 × 10^−3^) and dislocation density (8.48 × 10^15^) of the dose rate of 2.60 × 10^17^ ions/cm^2^·h, the finest grain size, the largest microstrain and the highest dislocation density of implanted samples can be achieved when the dose rate rises to 5.18 × 10^17^ ions/cm^2^·h, the effect of refining is 26.13%, and the increment of microstrain and dislocation density are 26.3% and 45.6%, respectively. Moreover, the Williamson–Hall plots are fitted linearly by taking βcosθ along the y-axis and 4sinθ or 4sinθ/Y_hkl_ or 4sinθ(2/Y_hkl_)^1/2^ along the x-axis. In all of the W-H graphs, it can be observed that some of the implanted samples present a negative and a positive slope; a negative and a positive slope in the plot indicate the presence of compressive and tensile strain in the material.

## 1. Introduction

The aero-engine main-shaft bearing is a crucial structure component in the aerospace industry; the stability and reliability will directly affect the in-service safety of the whole airplane [1]. The exceptional comprehensive performance includes factors such as a high strength at elevated temperature, high hardness, good wear resistance, excellent inherent anticorrosion resistance and extended fatigue life time [2,3,4]. As a promising bearing material, 8Cr4Mo4V steel has become increasingly popular and has been widely used in industrial and aerospace applications [5,6,7,8,9,10,11]. In recent years, with the rapid development of industrial and aerospace field, the more stringent requirements are put forward under the complex and harsh service environmental conditions. Extensive research has revealed that the bearing surface often exhibits failure such as the pitting, erosion and fatigue spalling which often occurs on the outermost surface of materials, thus bringing out the mechanical and physical properties’ decrease [12,13,14,15]. It will enable us to sustain a great economic loss and serious accidents, hence it is highly essential to improve the material comprehensive properties to meet the service requirements.

In this regard, in order to further enlarge the real applications of 8Cr4Mo4V bearing steel and meet their needs in severe working conditions, surface modification is necessary to improve the properties including the hardness, fatigue strength, wear and corrosion resistance [16,17,18,19,20,21,22]. Most of the surface-modification methods applied for bearing materials are based on the inner physical and chemical interactive reactions that produce optimized structures in order to improve their own performance [23,24,25,26,27,28,29,30]. Among the existing surface-modification methods, plasma immersion ion implantation is one of the environmentally friendly heat treatment and promising techniques [31,32,33,34,35]. Plasma immersion ion implantation has attracted increasing interest in recent decades both as a tool for basic materials research and as a novel technology for modifying the near-surface composition, structure and properties of materials and keeping the original properties of matrix materials. The ability to treat complex geometries’ parts is also an important point to be extensively applied for plasma immersion ion implantation. However, the existing plasma immersion ion implantation generally takes a much longer duration to achieve the required properties, thus causing the disadvantages of energy consumption and low implantation efficiency [36,37,38].

The nanoscale modified layer treated by ion implantation was determined by the implantation energy, dose and dose rate. Previous studies have revealed that the nanoscale modified layer differs from the bulk particles due to its unique physical and chemical properties [39,40,41]. In order to obtain a satisfied nanoscale modified layer, a high implantation dose range from 10^17^ to 10^19^ /cm^2^ is required during implantation process [42,43,44,45]. The higher the implantation dose required, the faster the implantation efficiency caused, the better the properties achieved and the lower the production cost. Hence, it is reasonable to infer that the high dose ion implantation process for bearing applications could be widely accepted in future real applications. The high-dose-rate plasma immersion ion implantation is developed based on plasma-based ion implantation and is conducted in this research. During high-dose-rate plasma immersion ion implantation, the high ion current density is applied to the samples; the implementation of high-dose ion implantation and the formation of the nanoscale modified layer occur in a much shorter time as compared to traditional plasma immersion ion implantation, thus leading to a significant increasing implantation efficiency and reducing energy consumption, and holding the potential for efficient industrial applications. Concurrently, the energy deposition elevates the substrate temperature and deepens the injection layer in the treating process. Yet the injection temperature influences how the dose rate affects the material’s microstructure and properties, leading to a peak. How to optimize the injection parameters and balance the efficiency and performance represents one of the current problems.

It is well known that the defects in microstructure are able to cause the full-width at the half of the maximum peak’s broadening and shift the Bragg’s angle peaks of the X-ray diffraction data as well [46,47,48]. For a comprehensive understanding of the influence of nanoscale modified layer properties, the lattice parameters need to be calculated properly. A relatively simple and powerful way to predict the crystallite size based on the width of the diffraction peak is using Scherrer equation according to the current understanding of X-ray diffraction patterns. The full-width at the half of the maximum peak’s broadening is considered as a result of the increase in lattice distortions and the decrease in crystallite size [49,50,51]. X-ray diffraction pattern analysis is a convenient and practical tool to measure the lattice parameters including crystallite size and microstrain; nonetheless, there are other useful methods to calculate the lattice parameters including TEM, size–strain plot and Williamson–Hall (W-H) models but not limited to them [52,53,54,55]. Among them, the Williamson–Hall approach is a reliable method to consider the effect of crystallite size and microstrain formation produced by the peak’s broadening of XRD [56,57,58]. It still holds an irreplaceable position in determining the crystallite size and dislocation distribution compared to other available methods.

After going through the extensive literature, it is observed that limited research can be found for analyzing the grain size, dislocation density distribution and microstrain of 8Cr4Mo4V steel by high-dose-rate plasma immersion ion implantation. Hence, the high-dose-rate treatment on plasma immersion ion implantation with nitrogen ions is conducted on 8Cr4Mo4V bearing steel, and the detailed XRD analysis is investigated by using Scherrer’s equation, and three various Williamson–Hall models are employed for estimating crystallite size, dislocation density and microstrain to determine the microstructural properties comprehensively. In this work, the authors have applied the three Williamson–Hall models on the 8Cr4Mo4V steel sample treated by high-dose-rate ion implantation, and these models hold more referenced and practical value in the present study; they are able to offer the experimental reference and theoretical guidance for more different materials. The current work exposes these approaches significantly in investigating the implanted crystallite size, lattice strain and dislocation density, and the experimental results will be useful for structural scientists and engineers and prompt the practical application of plasma immersion ion implantation in intelligent manufacturing.

## 2. Experimental

The material employed in this study is 8Cr4Mo4V bearing steel, with its chemical composition detailed in Table 1, supplied by Fushun Special Steel Co., Ltd, Fushun, China.

**Figure 1:**
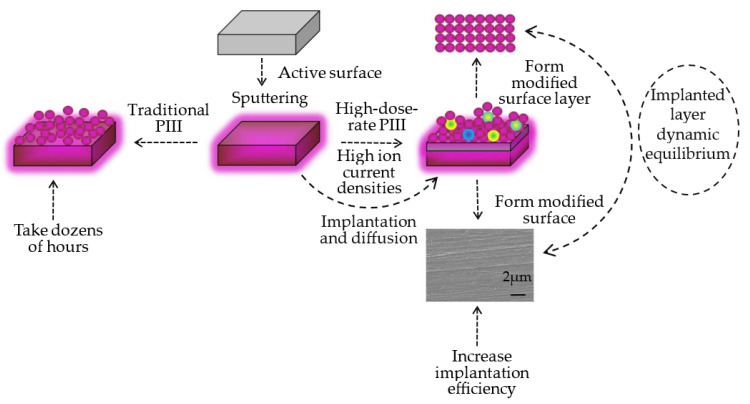
The schematic diagram of plasma immersion ion implantation process.

Before ion implantation, the 8Cr4Mo4V bearing steel was sectioned into flat-like samples with dimensions of 16.5 mm in diameter and 4.5 mm in thickness for structure analysis. The as-received samples were mechanically polished with a series of SiC abrasive papers with grits of 180# to 2000# and polished with diamond grit paste to a mirror finish. The samples were cleaned ultrasonically with acetone for 30 min before loading onto a sample holder in the DLZ-01 PIII facility which was described elsewhere [59]. The body of the chamber is connected with the grounded potential, and it acts as an anode. The sample holder is connected with the power supply, which acts as a cathodic plate. The oscilloscope and computer are connected with the equipment, which is able to monitor the voltage and current in the treating process in real time. The vacuum system was evacuated to a base pressure below 2.0 × 10^−3^ Pa and then back-filled with nitrogen to the working pressure of 0.3 Pa with a gas flow rate of 30 sccm. Ultimately, nitrogen ion implantations were conducted at ambient temperature and an acceleration energy of 20 keV at the same implantation dose (1.215 × 10^20^ ions/cm^2^, high dose) with various high dose rates (2.60 × 10^17^, 5.18 × 10^17^, 7.85 × 10^17^ and 1.04 × 10^17^ ions/cm^2^·h). In keeping with the premise of the same nitrogen ion implantation dose, we are able to control the dose rate through changes in the experimental parameters, including but not limited to the pulse width and frequency. The schematic diagram of the high-dose-rate plasma immersion ion implantation process is shown in Figure 1.

X-ray diffraction (XRD) with Cu-Kα radiation (λ = 1.54056 Å) was employed to investigate the phase composition of the nanoscale modified layer induced by nitrogen ion implantation.

## 3. Results and Discussion

### 3.1. SEM Images of the Modified Layer by High-Dose-Rate Ion Implantation

**Figure 2:**
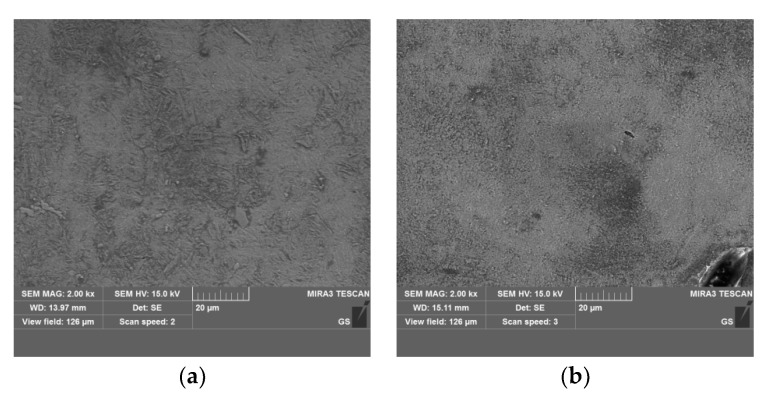
SEM images of a nanoscale modified layer treated under various implantation dose rates: (**a**) 2.60 × 10^17^ ions/cm^2^·h, (**b**) 1.04 × 10^18^ ions/cm^2^·h.

The SEM images of a nanoscale modified layer treated under various implantation dose rates are shown in Figure 2. It can be clearly observed that the samples implanted with a dose rate of 2.60 × 10^17^ ions/cm^2^·h exhibit a relatively smooth surface, while the implanted particles and holes from the SEM image are captured after implantation with a high dose rate of 1.04 × 10^18^ ions/cm^2^·h. This could be due to an additive increase in the elements’ sputtering coefficient with an increased number of atoms in the bombarding clusters during high-dose-rate plasma immersion ion implantation.

### 3.2. XRD Analysis of the Modified Layer by High-Dose-Rate Ion Implantation

**Figure 3:**
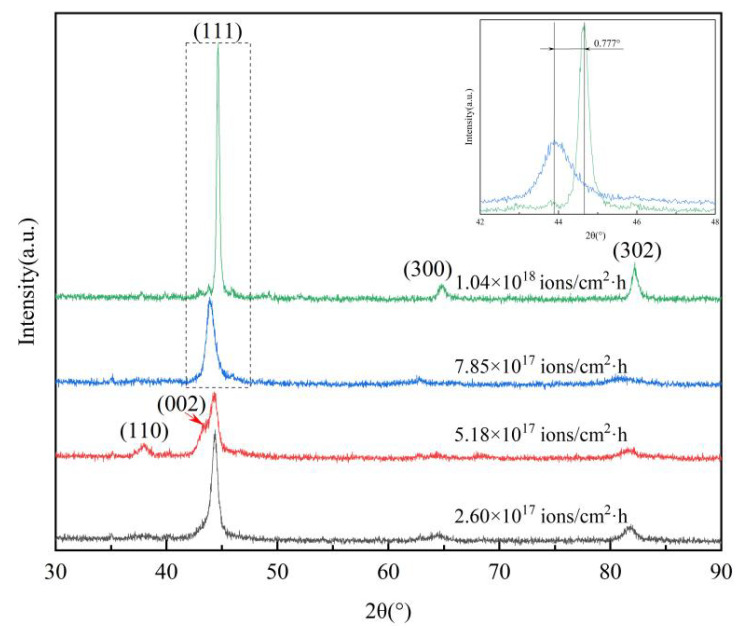
XRD patterns of samples treated under different implantation dose rates. (black: 2.60 × 10^17^ ions/cm^2^·h; red: 5.18 × 10^17^ ions/cm^2^·h; blue: 7.85 × 10^17^ ions/cm^2^·h; green: 1.04 × 10^18^ ions/cm^2^·h).

**Figure 4:**
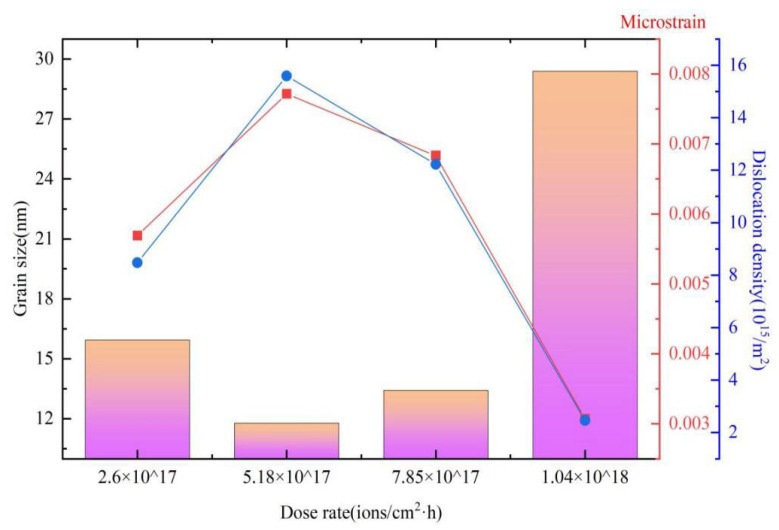
The plots of variation in crystallite size and microstrain dislocation density of samples treated under different implantation dose rates. (square: Grain size; red line: Microstrain; blue line: Dislocation).

The XRD patterns of a nanoscale modified layer treated under various implantation dose rates are shown in Figure 3. As can be observed from Figure 2, all of the implantation dose rates have three peaks, namely (111), (300) and (302), apart from the dose rate of 5.18 × 10^17^ ions/cm^2^·h which causes the formation of two extra peaks in the outermost implanted surface layer, as a consequence of the incorporation of iron and nitrogen atoms towards the matrix in a nonhomogeneous way, and forms the iron nitride compound phase. Meanwhile, the peaks corresponding to the implanted samples with a dose rate from 2.60 × 10^17^ to 7.85 × 10^17^ ions/cm^2^·h broadened and shifted slightly to the left by 0.777° compared with that of the implanted samples with a dose rate of 1.04 × 10^18^ ions/cm^2^·h, indicating that high-dose-rate ion implantation results in the fractional substitution of nitrogen ions at the iron site and causes an increase in lattice distortion in the implanted surface layer. The low peak shifting can also be ascribed to the fact that during the replacement of C with N, the crystallite size decreases, leading to a difference in the ionic radii of N and C. Peak shifting indicates a volume expansion of the unit cell; the lattice parameter for different dose rates has different values. Lattice expansion due to increased lattice parameters resulted in peak-shifting towards a lower angle, which indicates the lattice expansion of iron in 8Cr4Mo4V bearing steel due to N supersaturation, which was also confirmed by an increase in the lattice parameters as shown in Figure 4 [60]. For the samples implanted at the same dose, in addition to the inherent influence of ion implantation, the implantation dose rate plays an critical role in causing differences in the phase constituents as well. The higher-dose-rate implantation might act as a driving force for the broadening and shifting of diffraction peaks and the formation of phases to some extent. 

### 3.3. Determination of Crystallite Size by Scherrer Analysis

The appearance of XRD peak broadening is due to both the sample and instrumental common effects, while the increase in lattice size and microstrain is due to the dislocation [48,49]. To better understand their individual contributions, it is essential to collect a standard material diffraction pattern to determine the instrumental broadening precisely [61,62]. The corrected instrumental broadening *β*_hkl_ corresponds to the diffraction peak of the nanoscale modified layer can be measured by using the following equation [61,63]:

(1) $$\beta_{\mathrm{hkl}}^{}=\sqrt{\beta_{\mathrm{measured}}^{2}-\beta_{\mathrm{instrumental}}^{2}}$$

where *β*_measured_ and *β*_instrumental_ represent the measured and instrumental broadening, respectively. Generally, the crystallite size of the nanoscale modified layer is able to be estimated based on the XRD peak width through the following Scherrer equation [64,65,66,67]. The lattice strain and dislocation density induced in the modified layer are due to the crystal imperfection and distortion, which can be estimated by using the following formula [66,68]:

(2) $$D=\frac{k\lambda }{\beta_{\mathrm{hkl}}\cos\theta }$$

(3) $$\varepsilon =\frac{\beta }{4\tan\theta }$$

(4) $$\delta =16.1\frac{\varepsilon^{2}}{b^{2}}$$

where D represents the crystallite size (nm); k represents the shape factor, which here is 0.89; λ represents the wavelength of incident Cu-Kα, which here equals to 1.54056 Ǻ; β represents the peak width at half-maxima intensity; θ represents the peak position; ε is the microstrain; δ is dislocation density; and b is the Burgers vector.

The microstructural parameters containing the crystallite size, microstrain and dislocation density distribution of samples treated under various implantation dose rates are shown in Figure 4. It can be observed that the trend in crystallite size initially decreases and then tends to increase with the increase in dose rate. At the same time, it can be observed that the microstrain and dislocation density of the samples display the same regularities, namely they initially increase and then tend to decrease with the dose rate. At the dose rate of 5.18 × 10^17^ ions/cm^2^·h, both the microstrain and dislocation density reach the highest level of 7.716 × 10^−3^ and 1.559 × 10^16^ /m^2^, respectively.

The reason for this phenomenon is the incident nitrogen ions releasing energy into the samples and towards the matrix via sputtering and implanting, then the atoms begin the dynamic equilibrium in the implanted layer during the ion implantation process, which is shown vividly in Figure 1. Thus, the difference in the d-spacing of the crystal lattice planes results in the variation in the diffraction intensity [69]. It can be noticed that the transferred ion implantation energy reduces the microstrain along with the increase in crystallite size of the 8Cr4Mo4V steel sample at the cost of the grain boundaries [70]. As the crystallite size increases, its contribution to the observed peak width becomes smaller, which is demonstrated in X-ray diffraction analysis. The lattice distortions in the implanted 8Cr4Mo4V steel surface layer are another source of diffraction peak broadening. These can derive from the microstrain induced by compressive and tensile forces during high-dose-rate ion implantation, or they can arise from elements’ concentration gradients in the sample [71].

### 3.4. Estimation of Crystallite Size and Microstrain by Uniform Deformation Model (UDM)

From Equations (2) and (3), it can be clearly seen that the diffraction peaks’ broadening can be attributed to the crystallite size, and the broadening contributes to the microstrain. The relationship between the diffraction peaks’ broadening and lattice strain is given as Equation (5) [71,72]:

(5) $$\beta_{\mathrm{hkl}}=\frac{k\lambda }{D\cos\theta }+4\varepsilon \tan\theta$$

On rearranging Equation (5), we can obtain the following Equation (6) equivalently:

(6) $$\beta_{\mathrm{hkl}}\cos\theta =\frac{k\lambda }{D}+4\varepsilon \sin\theta$$

**Figure 5:**
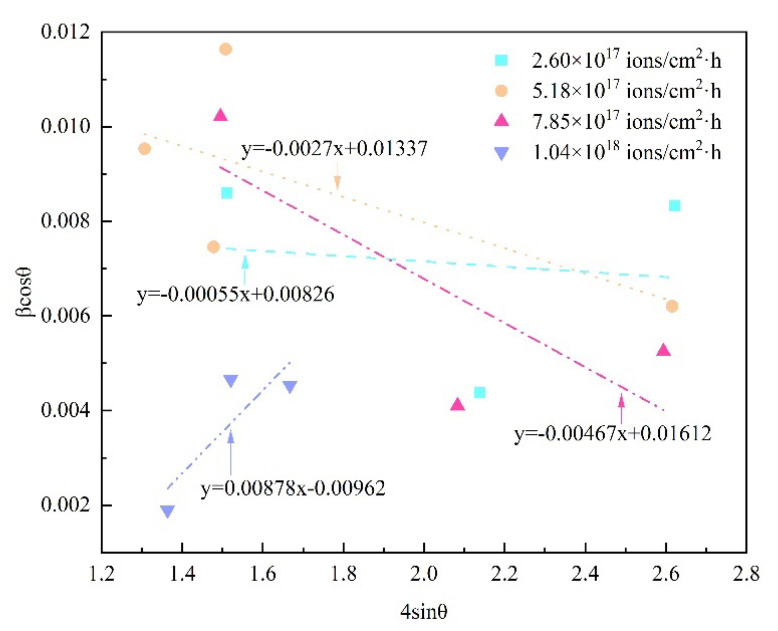
Williamson–Hall-UDM plots of 8Cr4Mo4V steel for various dose rates.

The above Equation (6) is an equation of a straight line and is regarded as a Williamson–Hall equation, which consider the isotropic nature of the crystals. In a Williamson–Hall-UDM model, the crystallite size and microstrain are assumed to be independent of each other. The Williamson–Hall–UDM plots of 8Cr4Mo4V steel for various dose rates are shown in Figure 5. As shown in Figure 4, it can be clearly seen that the plots are drawn with 4sinθ along the x-axis and β_hkl_cosθ along the y-axis, and the lines are in good accordance with Equation (6). The slope of the plotted straight line offers the lattice strain value, whereas the intercept gives the value of the crystallite size. The calculated slope of the line increases with the dose rate, and the slope value is greater for the implanted sample dose rate of 1.04 × 10^18^ ions/cm^2^·h than for the dose rate of 2.60 × 10^17^ ions/cm^2^·h. It is clear that there exists the same trend of initial increase then decrease in the lattice strain with increasing implantation dose rate, which corresponds well with the Scherrer results. Moreover, the negative and positive slope of Williamson–Hall-UDM plots can been found, which infers the presence of compressive and tensile strain in the modified layer of 8Cr4Mo4V steel under high-dose-rate nitrogen ion implantation.

### 3.5. Estimation of Crystallite Size and Lattice Deformation Stress by Uniform Stress Deformation Model (USDM)

The Williamson–Hall-UDM method is based on the valid assumption that the samples are homogeneous and isotropic in nature, which is not satisfied in many cases and therefore a new anisotropic method is proposed [67]. In the modified Williamson–Hall-USDM model, the lattice deformation stress is assumed to be uniform and the microstrain is assumed to be small enough [66,67,68]. 

According to the Hooke’s law equation, lattice deformation stress and microstrain can be expressed as σ = εY_hkl_, where σ represents stress, ε represents microstrain and Y_hkl_ is the anisotropic Young’s modulus. The modified Williamson–Hall equation is given as follows [73,74,75]:

(7) $$\beta_{\mathrm{hkl}}\cos\theta =\frac{k\lambda }{D}+4\sigma \frac{\sin\theta }{Y_{\mathrm{hkl}}}$$

where Y_hkl_ represents the Young’s modulus in the direction perpendicular to the set of the miller plane (hkl) and the equation for the cubic crystal lattice is given in Equation (8) [61,67,76]:

(8) $$\frac{1}{Y_{\mathrm{hkl}}}=S_{11}-2\left[(S_{11}-S_{12})-\frac{1}{2}S_{44}\right]\left[\frac{h^{2}k^{2}+k^{2}l^{2}+h^{2}l^{2}}{h^{2}+k^{2}+l^{2}}\right]$$

where *S*_11_, *S*_12_ and *S*_44_ show the elastic compliances of the cubic iron nitrides layer and their values are 13.2 × 10^−12^ Pa^−1^, −6 × 10^−12^ Pa^−1^ and 9.5 × 10^−12^ Pa^−1^, respectively.

**Figure 6:**
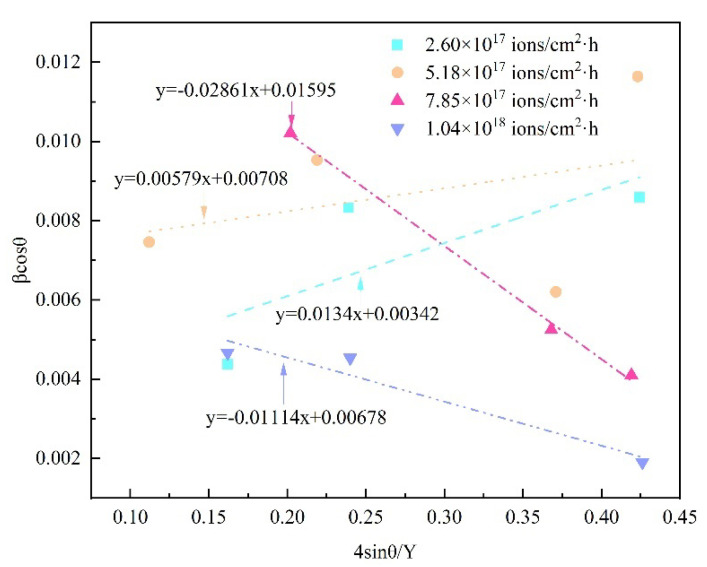
Williamson–Hall-USDM plots of 8Cr4Mo4V steel for various dose rates.

The typical plots of 8Cr4Mo4V steel for various dose rates between β_hkl_cosθ and 4sinθ/Y_hkl_ are given in Figure 6. The slope of the plotted straight line offers the value of the lattice deformation stress, whereas the intercept of the y-axis provides the average crystallite size of the nanoscale modified layer. As can be easily seen from the Williamson–Hall-USDM graph, it can be found that the stress calculated from the slope of the line is slightly smaller for the implanted sample with a dose rate of 1.04 × 10^18^ ions/cm^2^·h than for the dose rate of 2.60 × 10^17^ ions/cm^2^·h, and the slope of the line reaches its highest for the implanted sample with a dose rate of 7.85 × 10^17^ ions/cm^2^·h. The higher the increase in the slope of the approximating straight line, the sharper the increase in the magnitude of the microdistortions. The results observed herein are consistent with the observation from the Williamson–Hall-UDM model. 

### 3.6. Estimation of Deformation Energy Density by Uniform Deformation Energy Density Model (UDEDM)

The lattice strain is assumed to be isotropic in the UDM model, whereas the deformation stress and strain is assumed to be anisotropic in the USDM model. Unfortunately, the linear relation between deformation stress and strain can be regarded as invalid due to the existence of crystal imperfections, dislocations and different defects. As a result, the strain energy density is assumed to be uniform and it is considered that the energy density is the source of anisotropic microstrain in the Uniform Deformation Energy Density Model (UDEDM).

According to Hooke’s law for the calculation of energy (u = ε^2^Y_hkl_/2), where u represents the deformation energy density, ε represents the lattice strain and Y_hkl_ is the anisotropic Young’s modulus. Substituting u into Equation (7) will obtain the modified Williamson–Hall equation to represent the UDEDM [52,68,74,75,76]:

(9) $$\beta_{\mathrm{hkl}}\cos\theta =\frac{k\lambda }{D}+4\sin\theta {\left(\frac{2u}{Y_{\mathrm{hkl}}}\right)}^{1/2}$$

**Figure 7:**
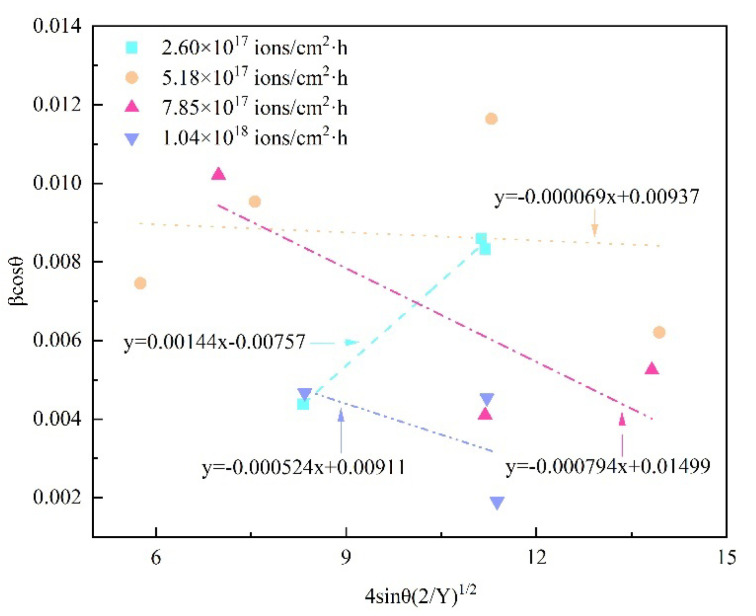
Williamson–Hall-UDEDM plots of 8Cr4Mo4V steel for various dose rates.

The plots of 8Cr4Mo4V steel for various dose rates between 4sinθ(2/Y_hkl_)^1/2^ (x-axis) and β_hkl_cosθ (y-axis) are given in Figure 7. As can clearly be seen from Figure 6, the crystallite size and anisotropic deformation energy density are determined by the intercept of the y-axis and the strain by the slope of the x-axis. By using the above-mentioned equation, the values of deformation energy density, crystallite size and lattice strain can be calculated at the same time. It can be observed that the slope value of the line is much smaller for the implanted sample with a dose rate of 2.60 × 10^17^ ions/cm^2^·h than for the dose rate of 1.04 × 10^18^ ions/cm^2^·h, and the slope value reaches its highest for the implanted sample with a dose rate of 7.85 × 10^17^ ions/cm^2^·h. Furthermore, it is worth noting that there exists a negative and positive slope in the plots, which indicates the presence of compressive and tensile strain in the modified layer of 8Cr4Mo4V steel under high-dose-rate nitrogen ion implantation [53,68,77]. The combined increase in lattice distortion at different positions in the implanted surface layer resulted in the diffraction peak broadening. The three various Williamson–Hall models have been proven to be the effective methods for understanding the microstructure evolution of 8Cr4Mo4V steel under high-dose-rate ion implantation and estimating the lattice parameters of materials.

## 4. Conclusions

In the present study, an attempt to understand the influence of plasma immersion ion implantation on the modified layer of 8Cr4Mo4V bearing steel in various dose rates of 2.60 × 10^17^, 5.18 × 10^17^, 7.85 × 10^17^ and 1.04 × 10^18^ ions/cm^2^·h has been investigated. The main conclusions obtained are drawn as follows:

(1) X-ray diffraction analysis suggests that the peaks corresponding to the implanted samples with a dose rate from 2.60 × 10^17^ to 7.85 × 10^17^ ions/cm^2^·h broadened and slightly shift to left by 0.777° towards, when compared with that of the implanted samples with a dose rate of 1.04 × 10^18^ ions/cm^2^·h.
(2) The crystallite size initially decreased and then tended to increase; the microstrain and dislocation density initially increased and then tended to decrease with the increasing of dose rate. The microstrain and dislocation density reached the highest level of 7.716 × 10^−3^ and 1.559 × 10^15^ /m^2^, respectively, at the dose rate of 5.18 × 10^17^ ions/cm^2^·h.
(3) In all of the Williamson–Hall graphs, the slope of the line reveals the different angle and values, which implies that the higher the increase in the slope of the approximating straight line, the sharper the increase in the magnitude of the microdistortions.
(4) Williamson–Hall methods reveal that there exist a negative and positive slope in various orientations, indicating a compressive and tensile strain induced in 8Cr4Mo4V steel by various high dose rates during plasma immersion ion implantation.
(5) The Williamson–Hall methods in this work demonstrate the feasibility of calculating lattice parameters and provide a further application for different materials to estimate the crystallite size, microstrain and dislocation density.

## Figures and Tables

**Table 1:** Chemical composition of 8Cr4Mo4V steel (wt.%).

C	Cr	Mo	V	Ni	Mn	Si	Co	W	Fe
0.8~0.85	4.0~4.25	4.0~4.5	0.9~1.1	≤0.15	0.15~0.35	≤0.25	≤0.25	≤0.25	Bal.

## Data Availability

The datasets used and/or analyzed during the current study available from the corresponding author on reasonable request.
